# Adverse Pregnancy Outcomes Following COVID-19 Infection or Vaccination in Active Component U.S. Military Service Women, 2021–2023

**Published:** 2025-10-01

**Authors:** Susan J. Ching, Jessica H. Murray, Natalie Y. Wells, Shauna L. Stahlman

**Affiliations:** Lackland Trainee Health Squadron, Joint Base San Antonio-Lackland, TX: Maj Ching; Armed Forces Health Surveillance Division, Public Health Directorate, Defense Health Agency, Silver Spring, MD: Ms. Murray, Dr. Wells, Dr. Stahlman

## Abstract

Prior studies have found a higher risk of adverse pregnancy outcomes due to COVID-19 infection; however, recent literature documents few adverse impacts to younger and otherwise healthy populations, but with limited information about military members. The study population comprised active component service women with a singleton delivery between 2021 and 2023. Adverse pregnancy outcomes were evaluated by COVID-19 infection and vaccination history, as well as by demographics and pre-existing comorbidities. During the surveillance period, 39,355 active component U.S. service women had a singleton delivery. After controlling for potential confounders in the adjusted logistic regression analysis, COVID-19 infection during pregnancy was associated with eclampsia (OR 2.18, *p* <0.05) and antepartum hemorrhage (OR 1.11, *p* <0.05), and COVID-19 infection prior to the start of pregnancy was associated with antepartum hemorrhage (OR 1.18, *p* <0.05). In comparison, after adjustment, COVID-19 vaccination during pregnancy and prior to start of pregnancy was not associated with increased odds of any adverse pregnancy outcome in active component service women. COVID-19 vaccines are recommended for pregnant women by the American College of Obstetricians and Gynecologists and, previously, the U.S. Centers for Disease Control and Prevention.

COVID-19 infection during pregnancy has been associated with an increased risk of certain pregnancy complications such as pre-eclampsia and pre-term birth. ^1 , 2^ Severity of COVID infection may also play a role, as more severe infections have been more strongly linked to pre-term premature rupture of membranes. ^3^ The increased risk for stillbirth and pre-eclampsia could be due to inflammatory changes affecting the placenta, and the need for intensive care associated with severe disease could result in the increased rates of pre-term delivery. ^4^

In 1 cohort study of electronic health care records in southeastern Texas, COVID-19 infection before and during pregnancy were associated with spontaneous abortion. ^5^ Other studies, however, found no association between COVID-19 infection and risk of miscarriage. ^6^ One matched retrospective cohort study of over 170,000 pregnancies found a 12% higher risk for gestational diabetes following COVID-19 infection during the first 21 weeks of pregnancy. ^7^ This association could be due to inflammation increasing insulin resistance, damage to the pancreas, or shared risk factors for more severe COVID-19 infection. ^8 , 9^

In contrast, studies of COVID-19 vaccination in pregnant women have not revealed increased risk of adverse maternal or neonatal outcomes including stillbirth, pre-term birth, hypertensive disorders, congenital malformations, or other conditions due to vaccination. ^10 - 12^ In fact, some studies have indicated that COVID-19 vaccination during pregnancy can reduce risk of stillbirth and pre-term birth. ^13 , 14^ Consequently, during the pandemic the U.S. Centers for Disease Control and Prevention (CDC) recommended that all pregnant patients remain up-to-date with COVID-19 vaccines before and during pregnancy. ^15^ The American College of Obstetricians and Gynecologists also recommends that patients receive an updated COVID-19 vaccine or ‘booster’ at any point during pregnancy. ^16^

**What are the new findings?**

This analysis found no significant difference in adverse pregnancy outcomes among those who received a COVID-19 vaccine prior to delivery compared to women who did not, between 2021 and 2023. COVID-19 infection prior to start of pregnancy was associated with antepartum hemorrhage whereas COVID-19 infection during pregnancy was associated with eclampsia and antepartum hemorrhage.

**What is the impact on readiness and force health protection?**

The findings from this analysis suggest there is a benefit to vaccinating pregnant active component service women against COVID-19. There was no increased risk of these adverse pregnancy outcomes associated with receipt of a COVID-19 vaccine in this study population. In contrast, COVID-19 infection may be associated with increased occurrence of some adverse pregnancy events.

Healthy women infected with COVID-19 during pregnancy primarily experience mild illness with limited or no significant adverse effects on the mother or neonate. ^4 , 17^ Women in active duty military service must maintain physical fitness standards and represent a relatively young and healthy population; as a result, it would be expected that COVID-19 infection would not increase risk of adverse pregnancy outcomes, in most situations. The objective of this study was to evaluate associations between COVID-19 infection during pregnancy and certain adverse pregnancy outcomes in active component U.S. service women who had a delivery between 2021 and 2023, with a review of any change in this association for women who received a COVID-19 vaccine during or prior to their pregnancy start dates. This study focused on adverse conditions that would be coded in the maternal record, since data from neonatal medical records were not available.

## Methods

### Study population

This cross-sectional study used inpatient and outpatient direct and purchased care medical encounter records from the Defense Medical Surveillance System (DMSS). The study population included U.S. active component service women who had a singleton delivery, either live or still birth outcome, from January 1, 2021 through December 31, 2023. International Classification of Diseases, 10th Revision, Clinical Modification (ICD-10-CM) codes were used to determine singleton live (Z370) or still births (Z371). The first birth event in this surveillance period was used if a woman had multiple delivery events during the period. Deliveries were included if a woman was on active component duty during the 280 days preceding the delivery date. The pregnancy start date was calculated as the date 280 days prior to the delivery event.

### Outcomes

The outcomes for this study were specific adverse pregnancy events diagnosed within 280 days preceding the first singleton delivery event during the surveillance period. Outcomes included antepartum hemorrhage or threatened abortion (ICD-10: O20* or O46*), gestational diabetes (O24.4*), eclampsia (O15*), pre-eclampsia (O14*), pre-term labor or delivery (O60*), premature rupture of membranes (O42*), and stillbirth (Z37.1). For the gestational diabetes analysis, individuals were excluded from the study population if they had an inpatient or outpatient diagnosis of ICD-10: E10* (type 1 diabetes), E11* (type 2 diabetes), O24.4* (gestational diabetes), or O24.9* (unspecified diabetes) prior to the start of pregnancy.

### Exposures of interest

The exposures of interest in this study were COVID-19 infection before or during pregnancy and COVID-19 vaccination before or during pregnancy. The Armed Forces Health Surveillance Division (AFHSD) maintains a master list of COVID-19 cases for active component service members. These COVID-19 cases were identified from reports of positive antigen, polymerase chain reaction (PCR), and confirmed or probable tests that were entered into the Disease Reporting System internet (DRSi) prior to January 2023, and Electronic Surveillance System for the Early Notification of Community-based Epidemics (ESSENSE) positive antigen and PCR tests that occurred on or after January 2023.

Anyone with multiple positive COVID-19 tests or reports was counted as 1 infection if both tests were within a 90-day period, consistent with guidelines from the CDC. ^18^ A woman was categorized as having a COVID-19 infection during pregnancy if there was a documented COVID-19 infection within 280 days prior to her delivery event, and categorized as having COVID-19 infection prior to pregnancy if it occurred more than 280 days prior to the delivery event.

DMSS immunization data were utilized to determine COVID-19 vaccination status. A single dose of any of the following CVX codes met criteria for receiving a COVID-19 vaccination: 207, 208, 212, 221, 217, 211, 229, 300, 309, 312, 313, 510, 511, 502, or 210. ^19^ These data are provided to DMSS from the MHS Information Platform (MIP) Immunizations Tracking System. DMSS only receives immunizations data for U.S. military service members.

### Covariates

Covariates for this study included age, race and ethnicity, service branch, number of prior deliveries, and comorbidities diagnosed prior to the start of the pregnancy. Electronic Periodic Health Assessment (PHA) data were reviewed to determine a service member*s self-reported smoking status within the 2 years prior the start of the associated pregnancy. Smoking was used as a covariate for its documented link to adverse maternal health outcomes. ^13^ Other lifestyle factors were not readily available from the PHA and thus were not included for covariate analysis. Pre-existing comorbidities were identified by having a diagnosis of that condition in any diagnostic position of an inpatient or outpatient encounter within 2 years prior to the delivery date **(Table 1)** . For assessment of the number of prior deliveries, 1 delivery was counted every 280 days (ICD-10 codes Z37*, O80, O82). All births were classified as vaginal or cesarian section (ICD-10: O82* or inpatient procedure codes 10D00; outpatient CPT codes 59510, 59515, 59514, 00850, 00857, 01961, 01963, 01968, 01969; diagnostic group codes 370, 371). Age, race and ethnicity, and service branch were assigned based on demographic data for the member at the time of the delivery event.

**TABLE 1.** ICD-10-CM Codes Utilized to Define COVID-19 Comorbidities

COVID-19 Comorbidities	ICD-10 Codes
Any lung disease	J40 * –J99 *
Any cardiovascular disease	I05 * –I89 * , Z95 *
Asthma	J45 *
Chronic kidney disease	N03 * –N16 * , N18 * –N19 *
Chronic liver disease	K70 * –K77 * , B18 *
Chronic lower respiratory disease	J40 * –J44 *
Chronic neurological disorders	G10 * –G40 *
Immune-compromising conditions	B20, D55 * -D77 * , D80 * –D89 * , Z94 * , Z795 * , L40 * , M04 * –M08 * , K50 * –K52 *
Metabolic disease	E08 * –E13 * , O24 * , Z794 * , E00 * –E07 * , E50 * –E64 * , E84 * , E88.81
Mood disorders, depression, schizophrenia	F20 * , F30 * -F39 *
Neoplasms	C00 * –D49 *
Obesity	E66.0 * , E66.1, E66.2, E66.3, E66.8, E66.9, Z68.3 * , Z68.4 * , O9921 *
Substance use disorders including nicotine dependence	F10 * –F16 * , F17 * , F18 * –F19 *
Tuberculosis	A15 *

Abbreviations: ICD-10-CM, International Classification of Diseases, 10th Edition, Clinical Modification; COVID-19, coronavirus disease 2019.

* Indicates all child codes included.

### Statistical analysis

Pearson chi-square tests were used to assess the relationship between exposures of interest and covariates with the adverse pregnancy outcomes. Adjusted logistic regression models were used to further explore the associations between the exposures of interest and study outcomes that were significant in the crude (unadjusted) analysis. These models adjusted for COVID-19 infection prior to the start of pregnancy, COVID-19 infection during pregnancy, COVID-19 vaccination prior to the start of pregnancy, COVID-19 vaccination during pregnancy, age, race and ethnicity, number of prior deliveries, and any previously diagnosed comorbidity. Covariates were selected for inclusion in the adjusted models based on being exposures of interest or significant potential confounders.

## Results

A total of 39,355 active component service women experienced a singleton delivery between January 1, 2021 and December 31, 2023 **(Table 2)** . Of those service women, 29,927 (76.0%) had vaginal deliveries, and 9,428 (24.0%) had cesarean sections. A total of 5,190 (13.2%) of these women had a documented COVID-19 infection during pregnancy, and 6,491 (16.5%) had a COVID-19 infection prior to pregnancy. Among women with an infection prior to the start of pregnancy, the first infection was a median of 233 days (IQR 110-402 days) prior.

**TABLE 2.** Demographics of Active Component U.S. Service Women with Singleton Births, 2021–2023

Demographics	Total	
	No.	%
Total	39,355	100
COVID-19 infection during pregnancy		
Yes	5,190	13.2
No	34,165	86.8
COVID-19 infection prior to start of pregnancy		
Yes	6,491	16.5
No	32,864	83.5
COVID-19 vaccination during pregnancy		
Yes	9,263	23.5
No	30,092	76.5
COVID-19 vaccination prior to start of pregnancy		
Yes	22,056	56.0
No	17,299	44.0
Age, *y*		
<20	655	1.7
20–24	13,868	35.2
25–29	12,087	30.7
30–34	8,239	20.9
35–39	3,862	9.8
40+	644	1.6
Race and ethnicity		
White, non-Hispanic	16,351	41.6
Black, non-Hispanic	8,953	22.8
Hispanic	8,784	22.3
Other	4,509	11.5
Unknown	758	1.9
Service branch		
Army	13,522	34.4
Navy	11,183	28.4
Air Force, Space Force	11,020	28.0
Marine Corps	2,777	7.1
Coast Guard	853	2.2
Comorbidities prior to pregnancy start date		
Cardiovascular disease	2,144	5.5
Chronic lower respiratory disease	318	0.8
Asthma	910	2.3
Lung disease	1,621	4.1
Metabolic disease	4,360	11.1
Immune compromising conditions	4,414	11.2
Substance use disorders (including nicotine dependence)	2,025	5.2
Chronic liver disease	217	0.6
Chronic kidney disease	1,003	2.6
Chronic neurological disorders	318	0.8
Neoplasms	3,749	9.5
Obesity	4,613	11.2
Tuberculosis	19	0.1
Mood disorders, depression, schizophrenia	3,891	9.9
Tobacco use (reported on PHA)	4,727	12.0
Prior deliveries, *n*		
0	27,189	69.1
1	9,023	22.9
2+	3,143	8.0

Abbreviations: No., number; COVID-19, coronavirus disease 2019; *y* , years; PHA, Periodic Health Assessment; *n* , number.

A total of 9,236 (23.5%) active component service women received at least 1 COVID-19 vaccine dose during pregnancy, and 22,056 (56.0%) received a dose prior to start of pregnancy. There were 27,685 (70.3%) women who received a vaccine dose on or prior to the delivery event, less than the sum of women (n=31,292) who received at least 1 dose during and prior to start of pregnancy, because some women received a dose both prior to and during pregnancy. The percentage of women who received at least 1 dose by their delivery date increased each calendar year: 30% for deliveries in 2021, 91% for deliveries in 2022, 99% for deliveries in 2023.

Most service women had no documented prior deliveries (69.1%). Most service women were ages 20–34 years (86.9%), while non-Hispanic White service women comprised the largest racial and ethnic group (41.6%). Obesity (11.7%), immunecompromising conditions (11.2%), and metabolic disease (11.1%) were the most commonly diagnosed comorbidities within the 2 years prior to pregnancy.

Without adjusting for any potential confounders, antepartum hemorrhage was the most common adverse pregnancy outcome (20.9%), followed by premature rupture of membranes (15.0%), gestational diabetes (8.3%), pre-eclampsia (8.2%), preterm labor or delivery (7.2%), stillbirth (0.8%), and eclampsia (0.1%) **(Table 3)** . Black, non-Hispanic service women had the highest percentage of pre-eclampsia, eclampsia, antepartum hemorrhage, and stillbirth. Generally, prevalence of adverse pregnancy outcomes tended to be higher among service women with certain pre-existing comorbidities. For example, pre-eclampsia and antepartum hemorrhage were more prevalent among service women with cardiovascular disease. Pre-eclampsia, gestational diabetes, antepartum hemor-rhage, and stillbirth were more common among service women with obesity.

**TABLE 3.** Pregnancy Outcomes, Singleton Births, Active Component U.S. Service Women 2021–2023

Demographics	Gestational Diabetes			Pre-Eclampsia			Eclampsia			Premature Rupture of Membrane			Pre-Term Labor or Delivery			Antepartum Hemorrhage			Stillbirth		
	No.	%	*p* -value	No.	%	*p* -value	No.	%	*p* -value	No.	%	*p* -value	No.	%	*p* -value	No.	%	*p* -value	No.	%	*p* -value
Total	3,259	8.3		3,230	8.2		53	0.13		5,895	15.0		2,830	7.2		8,210	20.86		323	0.8	
COVID-19 infection during pregnancy																					
Yes	418	8.1	0.5597	424	8.2	0.9152	13	0.25	0.0146	808	15.6	0.2016	401	7.7	0.1090	1161	22.37	0.0041	45	0.9	0.6914
No	2,841	8.3		2,806	8.2		40	0.12		5,087	14.89		2,429	7.1		7,049	20.63		278	0.8	
COVID-19 infection prior to start of pregnancy																					
Yes	540	8.4	0.8917	554	8.5	0.2927	8	0.12	0.7836	1,036	16.0	0.0153	442	6.8	0.1929	1,533	23.62	<0.0001	56	0.9	0.6815
No	2,719	8.3		2,676	8.1		45	0.14		4,859	14.8		2,388	7.3		6,677	20.32		267	0.8	
COVID-19 vaccination during pregnancy																					
Yes	810	8.8	0.0589	742	8.0	0.4296	11	0.12	0.6328	1,384	14.9	0.9070	702	7.9	0.0987	1,860	20.08	0.0343	88	1.0	0.1148
No	2,449	8.2		2,488	8.3		42	0.14		4,511	15.0		2,128	7.1		6,350	21.10		235	0.8	
COVID-19 vaccination prior to start of pregnancy																					
Yes	1,845	8.4	0.4441	1,798	8.2	0.6514	29	0.13	0.8456	3,405	15.4	0.0040	1,463	6.6	<0.0001	4,606	20.88	0.9043	181	0.8	0.9981
No	1,414	8.2		1,432	8.3		24	0.14		2,490	14.4		1,367	7.9		3,604	20.83		142	0.8	
Age, *y*																					
<20	20	3.1	<0.0001	62	9.5	<0.0001	3	0.46	0.0547	128	19.5	<0.0001	72	11.0	<0.0001	163	24.89	<0.0001	6	0.9	0.0001
20–24	796	5.8		1,318	9.5		20	0.14		2,146	15.5		1,062	7.7		3,127	22.55		106	0.8	
25–29	971	8.1		951	7.9		14	0.12		1,875	15.5		824	6.8		2,393	19.80		83	0.7	
30–34	845	10.3		553	6.7		6	0.07		1,197	14.5		522	6.3		1,582	19.20		65	0.8	
35–39	530	13.8		277	7.2		8	0.21		484	12.5		296	7.7		803	20.79		50	1.3	
40+	97	15.3		69	10.7		2	0.31		65	10.1		54	8.4		142	22.05		13	2.0	
Race and ethnicity																					
White, non-Hispanic	1,205	7.4	<0.0001	1,290	7.9	<0.0001	22	0.13	0.0278	2,310	14.1	<0.0001	1,076	6.6	<0.0001	2,835	17.34	<0.0001	111	0.7	0.0007
Black, non-Hispanic	658	7.4		869	9.7		20	0.22		1,297	14.5		796	8.9		2,360	26.36		105	1.2	
Hispanic	769	8.8		661	7.5		7	0.08		1,446	16.5		588	6.7		1,988	22.63		71	0.8	
Other	561	12.5		353	7.8		2	0.04		748	16.6		310	6.9		882	19.56		32	0.7	
Unknown	66	8.7		57	7.5		2	0.26		94	12.4		60	7.9		145	19.13		4	0.5	
Service branch																					
Army	1,082	8.0	<0.0001	1,140	8.4	0.0005	29	0.21	0.0058	2,252	16.6	<0.0001	1,093	8.1	<0.0001	2,918	21.58	0.0468	117	0.9	0.6473
Navy	1,059	9.5		992	8.9		5	0.04		1,548	13.8		776	6.9		2,317	20.72		82	0.7	
Air Force, Space Force	889	8.1		850	7.7		16	0.15		1,572	14.3		718	6.5		2,257	20.48		93	0.8	
Marine Corps	151	5.5		191	6.9		3	0.11		392	14.1		201	7.2		563	20.27		26	0.9	
Coast Guard	78	9.2		57	6.7		0	0.00		131	15.4		42	4.9		155	18.17		5	0.6	
Comorbidities prior to pregnancy start date																					
Cardiovascular disease																					
Yes	192	9.1	0.1730	237	11.0	<0.0001	4	0.19	0.5004	261	12.2	0.0002	181	8.4	0.0211	534	24.91	<0.0001	24	1.1	0.1149
No	3,067	8.3		2,993	8.0		49	0.13		5,634	15.1		2,649	7.1		7,676	20.63		299	0.8	
Chronic lower respiratory disease																					
Yes	34	10.9	0.1006	25	7.9	0.8216	1	0.31	0.3800	47	14.8	0.9204	27	8.5	0.3677	90	28.30	0.0010	5	1.6	0.1358
No	3,225	8.3		3,205	8.2		52	0.13		5,848	15.0		2,803	7.2		8,120	20.80		318	0.8	
Asthma																					
Yes	89	9.9	0.0868	85	9.3	0.2076	1	0.11	0.8366	119	13.1	0.1038	59	6.5	0.4033	226	24.84	0.0028	8	0.9	0.8434
No	3,170	8.3		3,145	8.2		52	0.14		5,776	15.0		2,771	7.2		7,984	20.77		315	0.8	
Lung disease																					
Yes	162	10.1	0.0083	146	9.0	0.2311	3	0.19	0.5720	217	13.4	0.0666	131	8.1	0.1564	423	26.10	<0.0001	13	0.8	0.9319
No	3,097	8.2		3,084	8.2		50	0.13		5,678	15.1		2,699	7.2		7,787	20.64		310	0.8	
Metabolic disease																					
Yes	490	11.6	<0.0001	374	8.6	0.3444	8	0.18	0.3513	583	13.4	0.0016	360	8.3	0.0039	1,087	24.93	<0.0001	44	1.0	0.1436
No	2,769	7.9		2,856	8.2		45	0.13		5,312	15.2		2,470	7.1		7,123	20.35		279	0.8	
Immune compromising conditions																					
Yes	333	7.6	0.0694	355	8.0	0.6721	7	0.16	0.6457	583	13.2	0.0005	397	9.0	<0.0001	1,069	24.22	<0.0001	42	1.0	0.3067
No	2,926	8.4		2,875	8.2		46	0.13		5,312	15.2		2,433	7.0		7,141	20.44		281	0.8	
Substance use disorders (including nicotine dependence)																					
Yes	196	9.7	0.0192	197	9.7	0.0105	2	0.10	0.6510	330	16.3	0.0881	173	8.5	0.0156	502	24.79	<0.0001	20	1.0	0.3926
No	3,063	8.2		3,033	8.1		51	0.14		5,565	14.9		2,657	7.1		7,708	20.65		303	0.8	
Chronic liver disease																					
Yes	23	10.9	0.1719	15	6.9	0.4859	1	0.46	0.1889	33	15.2	0.9247	18	8.3	0.5279	60	27.65	0.0136	2	0.9	0.8688
No	3,236	8.3		3,215	8.2		52	0.13		5,862	15.0		2,812	7.2		8,150	20.82		321	0.8	
Chronic kidney disease																					
Yes	93	9.4	0.2307	89	8.9	0.4363	4	0.40	0.0209	145	14.5	0.6386	80	8.0	0.3296	229	22.83	0.1198	8	0.8	0.9345
No	3,166	8.3		3,141	8.2		49	0.13		5,750	15.0		2,750	7.2		7,981	20.81		315	0.8	
Chronic neurological disorders																					
Yes	37	11.6	0.0311	32	10.1	0.2261	1	0.31	0.3800	47	14.8	0.9204	31	9.8	0.0763	86	27.04	0.0064	2	0.6	0.7035
No	3,222	8.3		3,198	8.2		52	0.13		5,848	15.0		2,799	7.2		8,124	20.81		321	0.8	
Neoplasms																					
Yes	334	9.0	0.1347	299	8.0	0.5866	4	0.11	0.6234	494	13.2	0.0012	270	7.2	0.9782	844	22.51	0.0089	43	1.2	0.0199
No	2,925	8.2		2,931	8.2		49	0.14		5,401	15.2		2,560	7.2		7,366	20.69		280	0.8	
Obesity																					
Yes	625	13.7	<0.0001	448	9.7	<0.0001	7	0.15	0.7365	641	13.9	0.0282	347	7.5	0.3540	1,113	24.13	<0.0001	62	1.3	<0.0001
No	2,634	7.6		2,782	8.0		46	0.13		5,254	15.1		2,483	7.2		7,097	20.43		261	0.8	
Tuberculosis																					
Yes	3	16.7	0.1990	2	10.5	0.7126	0	0.00	0.8728	3	15.8	0.9211	2	10.5	0.5735	4	21.05	0.9836	0	0.0	0.6916
No	3,256	8.3		3,228	8.2		53	0.13		5,892	15.0		2,828	7.2		8,206	20.86		323	0.8	
Mood disorders, depression, schizophrenia																					
Yes	394	10.2	<0.0001	353	9.1	0.0384	10	0.26	0.0284	545	14.0	0.0734	360	9.3	<0.0001	969	24.90	<0.0001	40	1.0	0.1311
No	2,865	8.1		2,877	8.1		43	0.12		5,350	15.1		2,470	7.0		7,241	20.42		283	0.8	
Tobacco use (reported on PHA)																					
Yes	446	9.5	0.0021	450	9.5	0.0005	8	0.17	0.4896	735	15.6	0.2418	366	7.7	0.1174	1,061	22.45	0.0043	39	0.8	0.9720
No	2,813	8.2		2,780	8.0		45	0.13		5,160	14.9		2,464	7.1		7,149	20.65		284	0.8	
Prior deliveries, *n*																					
0	2,120	7.8	<0.0001	2,631	9.7	<0.0001	39	0.14	0.0076	4,560	16.8	<0.0001	1,883	6.9	0.0068	5,888	21.66	<0.0001	215	0.8	0.0015
1	802	9.0		430	4.8		5	0.06		1,014	11.2		692	7.7		1,701	18.85		65	0.7	
2+	337	10.8		169	5.4		9	0.29		321	10.2		255	8.1		621	19.76		43	1.4	

Abbreviations: No., number; COVID-19, coronavirus disease 2019; *y* , years; PHA, Periodic Health Assessment; *n* , number.

Note: These findings are not adjusted for any potential confounders

In many cases, there was not a significant ( *p* <0.05) difference in prevalence of adverse pregnancy outcomes in service women according to COVID-19 infection or vaccination status, with a few notable exceptions **(Table 3)** . COVID-19 infection during pregnancy was associated with a higher percentage of eclampsia and antepartum hemorrhage; COVID-19 infection prior to start of pregnancy was associated with a higher percentage of antepartum hemorrhage and premature rupture of members; COVID-19 vaccination during pregnancy was associated with lower percentage of antepartum hemorrhage; and COVID-19 vaccination prior to the start of pregnancy was associated with a higher percentage of premature rupture of membranes and a lower percentage of pre-term labor or delivery.

After controlling for potential confounders in the adjusted logistic regression analysis, COVID-19 infection during pregnancy remained significantly and positively associated with eclampsia (OR 2.18, *p* <0.05) and antepartum hemorrhage (OR 1.11, *p* <0.05), and COVID-19 infection prior to start of pregnancy remained significantly and positively associated with antepartum hemorrhage (OR 1.18, *p* <0.05) **(Table 4)** . After adjustment, COVID-19 vaccination prior to start of pregnancy was no longer associated with premature rupture of membranes. COVID-19 vaccination prior to start of pregnancy was, however, inversely associated (OR 0.86, *p* <0.05) with pre-term labor or delivery.

**TABLE 4.** Adjusted ^a^ Odds of Pregnancy Outcomes, Singleton Births, Active Component U.S. Service Women, 2021–2023

Exposure	Eclampsia			Premature Rupture of Membrane			Pre-Term Labor or Delivery			Antepartum Hemorrhage		
	OR	95% CI Lower Limit	95% CI Upper Limit	OR	95% CI Lower Limit	95% CI Upper Limit	OR	95% CI Lower Limit	95% CI Upper Limit	OR	95% CI Lower Limit	95% CI Upper Limit
COVID-19 infection during pregnancy												
Yes	2.18	1.16	4.10	1.06	0.98	1.15	1.09	0.98	1.22	1.11	1.03	1.19
No	Reference	—	—	Reference	—	—	Reference	—	—	Reference	—	—
COVID-19 infection prior to start of pregnancy												
Yes	0.95	0.43	2.07	1.06	0.98	1.14	0.98	0.88	1.10	1.18	1.10	1.26
No	Reference	—	—	Reference	—	—	Reference	—	—	Reference	—	—
COVID-19 vaccination prior to start of pregnancy												
Yes	0.92	0.52	1.63	1.03	0.97	1.09	0.86	0.79	0.93	0.96	0.91	1.01
No	Reference	—	—	Reference	—	—	Reference	—	—	Reference	—	—
COVID-19 vaccination during pregnancy												
Yes	0.85	0.43	1.68	1.05	0.98	1.12	1.06	0.97	1.16	0.98	0.92	1.04
No	Reference	—	—	Reference	—	—	Reference	—	—	Reference	—	—

Abbreviations: OR, odds ratio; CI, confidence interval; COVID-19, coronavirus disease 2019.

a Models included COVID-19 infection during pregnancy, COVID-19 infection prior to start of pregnancy, COVID-19 vaccination during pregnancy, COVID-19 vaccination prior to start of pregnancy, age, race and ethnicity, number of prior deliveries, and any previously diagnosed comorbidity

## Discussion

This study found increased odds of eclampsia and antepartum hemorrhage, which includes threatened abortion or any bleeding during pregnancy, among active component service women with a documented COVID-19 infection during pregnancy. In contrast, COVID-19 vaccination during or prior to start of a pregnancy was not associated with increased odds of any adverse pregnancy outcome, after adjustment for potentially confounding factors. It is important to note that these findings cannot be generalized to the U.S. population, nor to earlier periods during the COVID-19 pandemic when vaccines were not widely available, and pre-existing immunity was low or non-existent. It is possible that by the period of analysis for this study, members of the study population may have already had COVID-19 illness and thereby developed natural immunity, which could not be identified. It is estimated that by June 2021 74% of active component service members had been exposed to COVID-19, either by prior infection or vaccination. ^20^

A mandate issued by the U.S. Department of Defense (DOD) on August 24, 2021 required service members to receive a COVID-19 vaccination by December 31, 2021. That requirement was rescinded in January 2023, however, by Section 525 of the National Defense Authorization Act. ^21 , 22^ This study concurs with prior research that reveals that receipt of a COVID-19 vaccine prior to or during pregnancy was not associated with any change in adverse pregnancy outcomes, including antepartum hemorrhage and stillbirth. ^10 - 12^ The results of this study are also consistent with a recently published article from the DOD's Birth and Infant Health Registry, which found that COVID-19 vaccination was not associated with increased risk for pre-term birth, small size for gestational age, low birth weight, or neonatal intensive care unit admission among active duty service women who gave birth in 2021. ^23^

The highest percentage of adverse pregnancy outcomes occurred in Black, non-Hispanic service women, consistent with other research that reveals elevated levels of adverse pregnancy outcomes in this population. ^24^ This study population differs from most other studies, however, because military service women have access to robust medical care and surveillance during their pregnancies, which eliminates access to care as a potential confounder for the association between race and pregnancy outcome. Consequently, the findings in this study support the possibility of other factors besides access to care that contribute to the increased risk of adverse pregnancy outcomes in non-Hispanic Black service women. ^25^

Consistent with prior studies, pre-existing comorbidities were associated with different types of adverse pregnancy outcomes. ^26 - 28^ Further studies should be conducted to validate the finding of potential associations between COVID-19 infection and eclampsia and antepartum hemor-rhage, since it was not possible in this study to determine whether COVID-19 infection was the cause of those adverse outcomes.

Some limitations to this study are important to note. First, in this cross-sectional study design, temporality between COVID-19 infection or COVID-19 vaccination that occurred during pregnancy cannot be inferred with these adverse pregnancy outcomes. It is possible that some adverse pregnancy outcomes occurred prior to the documented COVID-19 infection. COVID-19 infection and vaccination prior to the start of pregnancy does infer temporality, however, which adds to the robustness of these findings.

Selection bias could have occurred in this study because pregnancies that ended in abortion, spontaneous or otherwise, were not included. If COVID-19 infection and adverse pregnancy outcomes are associated with spontaneous abortions, this would result in a negative bias, or an attenuation of the true association between COVID-19 infection and an adverse pregnancy outcome.

It is also unlikely that all COVID-19 infections during pregnancy were identified in this study, as at-home COVID test kits were rapidly deployed during the surveillance period. In addition, service women had the ability to test outside of the military's medical system, resulting possible in misclassification for some categorized as without COVID-19 infection when they were potentially infected during their pregnancy. Similarly, women with no or mild COVID-19 symptoms may not have realized they were infected and, therefore, would not have tested.

This study considered women with any dose of any COVID-19 vaccine as vaccinated. As such, a misclassification bias is possible if these women were not fully vaccinated. Remaining up-to-date with COVID-19 vaccines was believed to provide the most benefit in the prevention of both severe adverse pregnancy outcomes and COVID-19 disease. ^15^

Lastly, it should be noted that 52% (n=4,298) of the cases of antepartum hemorrhage had a diagnosis of O20.0 for “threatened abortion,” which can also be used to code bleeding during pregnancy. This coding could result in an over-estimate of antepartum hemorrhage cases, since bleeding during pregnancy is a more common and less severe outcome. Similarly, premature rupture of membranes may be over-estimated because 52% (n=3,079) of those cases had a diagnosis of O42.02, “Full-term premature rupture of membranes, onset of labor within 24 hours of rupture,” which suggests that, for half of these cases, the rupture occurred at or after 37 completed weeks of gestation.

This study provides insight on adverse pregnancy outcomes among pregnant U.S. active component service women. These findings suggest that COVID-19 vaccination is not associated with adverse pregnancy outcomes in this population. Future studies should review the prevalence of these outcomes in this population, refine and validate any associations with COVID-19 infection, along with the various levels of vaccination on adverse neonatal outcomes, and further investigate outcomes for pregnant active component service women of racial and ethnic minorities, to determine the reasons for these differences, given their equal access to no-cost medical care.
